# Characterization of *Fusarium* Spp. Inciting Vascular Wilt of Tomato and Its Management by a *Chaetomium*-Based Biocontrol Consortium

**DOI:** 10.3389/fpls.2021.748013

**Published:** 2021-11-19

**Authors:** Govindan Pothiraj, Zakir Hussain, Awani Kumar Singh, Amolkumar U. Solanke, Rashmi Aggarwal, Raman Ramesh, Veerubommu Shanmugam

**Affiliations:** ^1^ICAR-Indian Agricultural Research Institute, New Delhi, India; ^2^ICAR-National Institute of Plant Biotechnology, New Delhi, India; ^3^ICAR-Central Coastal Agricultural Research Institute, Goa, India

**Keywords:** *Fusarium* wilt, tomato, *Chaetomium*, *Trichoderma*, PGPR, consortium, biocontrol

## Abstract

Though the vascular wilt of tomato caused by the species of *Fusarium* is globally reported to be a complex disease in certain countries, for example, India, our studies indicated that the disease is caused by either *Fusarium oxysporum* f. spp. *lycopersici* (Fol) or *Fusarium solani* (FS) with the Fol being widely prevalent. In assessing the genetic diversity of 14 Fol strains representing the four Indian states by the unweighted pair group method with arithmetic averaging using Inter Simple Sequence Repeat (ISSR) amplicons, the strains distinguished themselves into two major clusters showing no correlation with their geographic origin. In pot experiments under polyhouse conditions, the seed dressing and soil application of a talc-based formulation of a biocontrol treatment, TEPF-Sungal-1 (*Pseudomonas putida*) + S17TH (*Trichoderma harzianum*) + CG-A (*Chaetomium globosum*), which inhibited Fol, was equally effective like the cell suspensions and was even better than the fungicidal mixture (copper oxychloride-0.25% + carbendazim-0.1%) in promoting the crop growth (52.3%) and reducing vascular wilt incidence (75%) over the control treatment, despite the challenge of inoculation with a highly pathogenic TOFU-IHBT strain. This was associated with significant expressions of the defense genes, indicating the induction of host resistance by a biocontrol consortium. In field experiments on two locations, the bioconsortium was highly effective in recording maximum mean fruit yields (54.5 and 60%) and a minimum mean vascular wilt incidence (37.5%) in comparison to the untreated control. Thus, *Chaetomium-*based bioconsortium demonstrated consistency in its performance across the two experiments in 2 years under the two field conditions.

## Introduction

Successful cultivation of tomato (*Lycopersicon esculentum* Mill) is limited by the vascular wilt disease caused by *Fusarium oxysporum* f. spp. *lycopersici* (Fol) W. C. Synder and H. N. Hans (Sidharthan et al., 2018, 2019). The disease inflicts a substantial yield loss ranging from 25 to 55% in many tomato growing areas of the country (Nirmaladevi et al., 2016) besides deteriorating the quality of fruits. Management practices have little impact on vascular wilt incidence mainly due to highly resistant chlamydospores produced by the pathogen under unfavorable conditions (Shanmugam and Kanoujia, 2011) and limited studies on the genetic variability of the fungal pathogen (Akbar et al., 2018). The soil application of fungicides besides failing to control the disease (Nirmaladevi et al., 2016) under field conditions, the appearance of fungicide-resistant pathogen strains, toxicity, and negative environmental effects of fungicides are of great concern (Shanmugam and Kanoujia, 2011). Although resistant tomato cultivars bearing major gene (*I/I-1, I-2*, and *I-3*) resistance to *Fusarium* wilt have been developed elsewhere (Huang and Lindhout, 1997) in India, no resistant varieties have been reported. Hence, the biological management of the disease could be a potential and attractive alternate management strategy that needs to be exploited.

In the biomanagement of *Fusarium* wilt of tomato, the potential of some plant-growth promoting rhizobacteria microbes such as the species of *Bacillus, Pseudomonas*, and *Trichoderma* has been reported under glasshouse conditions (Shanmugam et al., 2011a,b, 2015; Shanmugam and Kanoujia, 2011). Of late, the exploration of new biocontrol agents with an ample potential for disease control in plants leads to the identification of several species of *Chaetomium* as useful bioagents (Aggarwal et al., 2014). The filamentous fungi belonging to the class Ascomycetes and the family Chaetomiaceae usually survive as saprophytes in organic compost or soil or as endophytes in host plants (Aggarwal et al., 2008). Though some species are exploited in biotechnological industries for the case of being good laccase and cellulose producers (Abdel-Azeem et al., 2016), the species *Chaetomium globosum* has largely been identified as a potential biocontrol agent against damping off and several seed and soil borne diseases. Like other biocontrol agents, the biocontrol mechanisms in *C. globosum* are attributed to a competition for nutrients and substrates, mycoparasitism, antibiosis, or their combinations (Zhang and Yang, 2007). Besides exerting a direct antifungal activity, the biocontrol fungus is also known to induce systemic resistance in crop plants (Biswas et al., 2000; Aggarwal et al., 2016). The induction of defense enzymes has mostly been implicated in such plant-mediated resistance (Soytong et al., 2001). Despite the exploitation of *C. globosum* in biocontrol, its potential for the biomanagement of *Fusarium* wilt of tomato has not been reported.

Though the biological management of plant diseases using a single biocontrol agent is effective under controlled conditions, the performance of the bioagents is inconsistent in the field due to complex soil environments (Shanmugam and Kanoujia, 2011). The use of a microbial consortium is a potential alternative approach for the uncertainty experienced in biocontrol under field conditions. The strains constituting the consortia need to be carefully selected in such a way that they express different plant-growth promoting and biocontrol traits and that they complement each other to express the traits together (Shanmugam et al., 2013). Microbial consortia that perform better than individual strains in improving crop productivity by promoting plant growth directly or indirectly through their effects on the pathogen have been well documented in several crops (Shanmugam et al., 2011a,b, 2013; Shanmugam and Kanoujia, 2011). Several of these microbial consortia mainly constituted the species of *Bacillus, Pseudomonas, Serratia*, *Penicillium*, and *Trichoderma* (Shanmugam et al., 2013; Yang et al., 2014). The use of *C. globosum* for the biomanagement of plant diseases has mostly been resorted to using a single strain (Aggarwal, 2015) and not in a consortium. Microbial consortia consisting of antagonistic bacteria and fungi have an added advantage of protecting the plants under different conditions or at different times by occupying complementary or different niches (Larkin and Fravel, 1998).

In the present study, we, therefore, aimed to characterize the Fol inciting the vascular wilt of tomato to understand its genetic variability and to develop and test carrier-based formulations of microbial consortia comprising antagonistic *Bacillus subtilis* (S2BC-1), *Pseudomonas putida* (TEPF-Sungal-1), *Trichoderma harzianum* (S17TH), and *C. globosum* (CG-A) in varying combinations under glasshouse conditions. The induction of resistance in the crop plant by the consortium was assessed based on the expression of candidate defense genes. The potential consortium identified from the studies was then validated under field conditions for *Fusarium* wilt control.

## Materials and Methods

### Pathogen Isolates

Field surveys were carried out in the tomato fields of IARI Farm and National Capital Region (NCR) along the Yamuna plain (six locations) during 2017–2018, and *Fusarium-*infected plant samples were collected from each of the locations. The isolation of the fungus was made on a potato dextrose agar (PDA) medium from the infected tissues by the direct plating method as described earlier (Shanmugam et al., 2013). These isolates together with putative *Fusarium* isolates obtained from vascular wilt-infected tomato samples and available in the culture collections of the laboratory were used for these studies. The isolates were purified by a single spore isolation method, and their morphological characters (Nelson et al., 1983) were assessed to establish their identities. For pathogenicity assay, a conidial suspension was prepared as described earlier (Shanmugam et al., 2013) by pouring 20 ml of sterile tap water onto 7-day-old culture of each of the isolates. The aerial mycelium was then gently scraped using a sterile glass slide, and the fungal suspension was filtered using a sterile double-layer muslin cloth. The suspension was quantified with a hemocytometer, and the spore density was adjusted to 1 × 10^4^ conidia ml^–1^ with sterile tap water. The inoculation of the spore suspension was performed on 4-week-old tomato (cv. Pusa Rohini) plants maintained in a polyhouse by dipping the wounded roots in the conidial suspension for 10 min. Tomato plants dipped in sterile tap water served as control. The treated seedlings were then planted in 10-cm plastic pots containing 1.5 kg sterile soil. The wilt incidence was determined as percent disease index (PDI) using the scale described earlier (Shanmugam et al., 2011a) as 0 = no symptoms; 1 ≤ 25% of the leaves with symptoms; 2 = 26–50% of the leaves with symptoms; 3 = 51–75% of the leaves with symptoms; and 4 = 76–100% of the leaves with symptoms. The PDI was calculated as follows: Disease index = [Σ(rating × number of plants rated)/Total number of plants × highest rating] × 100. The pathogenic fungi were then reisolated from the diseased tissues exhibiting vascular browning by plating them on PDA after surface sterilization (1% sodium hypochlorite) and Koch’s postulates were confirmed. The fungus inciting a maximum wilt incidence was rated as a highly pathogenic isolate. The identity of the pathogenic isolates was reconfirmed by PCR amplification and partial sequencing of the Internal Transcribed Spacer (ITS) region and transcription elongation factor (*tef*) gene from the genomic DNA as described earlier (Divakara et al., 2014; Sidharthan et al., 2018). To isolate the genomic DNA, two individual colonies of each of the fungal isolates grown on PDA plates were inoculated separately in 100 ml of potato dextrose broth (PDB). The inoculated flasks were incubated on a rotary shaker at 28 ± 2°C and 180 rpm for 5 days. The mycelium was filtered out on a sterile Whatman No. 1 filter paper, washed three times with sterile distilled water to remove traces of the medium, and air-dried. The dried mycelial mat was ground with a mortar and pestle in liquid nitrogen, and the DNA was isolated from the ground mycelium by a commercial kit (Sigma, Burlington, MA, United States) following the manufacturer’s protocol. For the molecular characterization of the pathogen isolates, a region of the nuclear rRNA gene containing ITS regions 1 and 4 and the 5.8S rRNA gene was amplified from the genomic DNA by PCR using the primers, ITS1 (5′-TCCGTAGGTGAACCTGCGG-3′) and ITS4 (5′-TCCTCCGCTTATTGATATGC-3′) to get an amplicon size of 500–550 bp. PCR amplification of the *tef* gene (600 bp) from the genomic DNA was done with the primers, EF-F [5′-ATGGGTAAGGA(A/G)GACAAGAC-3′] and TEF-R [5′-GGA(G/A)GTACCAGT(G/C)ATCATGTT-3′]. The amplification of either of the genes was carried out in a 50 μl reaction volume consisting of 10X buffer, 5.0 μl; 2 mM dNTPs, 2.0 μl; 3 U/μl *Taq* DNA polymerase, 1.0 μl; 100 ng/μl of each primer, 2 μl; 50–100 ng template DNA, 3 μl; and H_2_O 32.0 μl in a Biorad thermal cycler using the PCR conditions of 94°C for 4 min (initial denaturation), and 35 cycles of 94°C for 1 min (denaturation), 55°C for 1 min (annealing), and 72°C for 2 min (extension). The PCR product was purified, was cloned into a pGEM-T easy vector (Promega, Madison, WI, United States) following the manufacturer’s protocol, and was sequenced. The deduced sequences were evaluated for a homology with the NCBI database, and the sequences were deposited in GenBank. The pathogenic isolates were stored as 15% glycerol stocks at −80°C in the laboratory culture collections.

### Genetic Diversity of Fol Strains

The deduced sequences of the ITS region were aligned using the LALIGN program and were grouped based on the levels of similarities in the consensus sequences. PCR amplifications of the Inter Simple Sequence Repeat (ISSR) region of genomic DNA were done with the primers, ISSR9 5′-GAGAGAGAGAGAGAGAGAC-3′, and ISSR10 5′-GAGAGAGAGAGAGAGAGAT-3′ (Nirmaladevi et al., 2016) and UBC855 (5′-ACACACACACACACACYT-3′ and UBC 856 (5′-ACACACACACACACACYA-3′) (Lin et al., 2012) in a Applied Biosystems^TM^ A24811 thermocycler using the conditions of 94°C for 5 min, 94°C for 30 s, 50°C for 1 min, and 72°C for 2 min for 35 cycles, with a final extension of 72°C for 10 min. The reaction mixture (25 μl) consisted of 14.5 μl of MilliQ water, 2.5 μl of 10X PCR buffer, 0.5 μl of 10 mM dNTPs, 2 μl of primer, 0.3 μl of 3 U of *Taq* DNA polymerase, and 100 ng genomic DNA as the template. The reaction mixture without the genomic DNA was served as the control. The PCR mixture was electrophoresed in 1% agarose gel, and the bands appearing consistently in the three PCR amplifications were evaluated.

### Biocontrol Strains and Evaluation for Antagonism

Four biocontrol strains, S2BC-1 (*B. subtilis*, GenBank No. AM268039), TEPF-Sungal-1 (*P. putida*, GenBank No. MZ363827), S17TH (*T. harzianum*, GenBank No. GU048855), and CG-A (*C. globosum* GenBank No. AY429049) available in the culture collections of the laboratory were used for these studies. The antifungal activity of the biocontrol strains against the fungal pathogen was established by a dual-culture assay on PDA (Shanmugam et al., 2011a,b). The biocontrol strains were either inoculated after 48 h of pathogen inoculation (S2BC-1 and TEPF-Sungal-1) or co-inoculated (S17TH and CG-A) with the pathogen on the same day in triplicate. PDA plates inoculated with the pathogen alone served as control. Upon incubating at 28°C ± 2°C and when the control plate displayed a full growth, the zone of inhibition between the colony margins was measured. A mutual inhibition among the biocontrol strains was also assessed by the dual-culture assay in triplicate.

### Preparation of Carrier-Based Formulations of Antagonistic Strains

The biocontrol strains and strain mixtures were mass multiplied in talc-based formulations to contain 1 cfu g^–1^ × 10^8^ cfu g^–1^ and 3 cfu g^–1^ × 10^6^ cfu g^–1^ of antagonistic bacteria and fungi, respectively (Shanmugam et al., 2011b). Briefly, the bacterial inoculum was prepared by suspending the cells (3 cfu ml^–1^ × 10^9^ cfu ml^–1^) grown in 150 ml of nutrient (S2BC-1) or King’s B (TEPF-Sungal-1) broth at 28°C ± 2°C on a rotary shaker for 48 h and collected by centrifugation at 6,000 *g* for 15 min at 4°C in 0.2 M sodium phosphate buffer (pH 7.0). The fungal inoculum was prepared by suspending the spores (3 cfu ml^–1^ × 10^9^ cfu ml^–1^) collected from 150 ml of potato dextrose broth after separating the mycelium by passing the inoculum through a muslin cloth. The broth was earlier inoculated with a mycelial disk (5 mm) of S17TH or CG-A and incubated at 28°C ± 2°C on a rotary shaker for 10 days. To prepare the inoculum for strain mixtures, the designated strains were cultured on their respective media as described, and equal volumes (v/v) of the strains were used. The carrier-based formulation for each of the treatments was then prepared by aseptically mixing 400 ml of the inoculum with autoclaved (121°C for 20 min for 2 consecutive days) talc powder (1 kg) containing 15 g CaCO_3_ and 10 g carboxymethyl cellulose (CMC). The moisture content in the product was reduced to less than 20% by shade drying before storage at 28 ± 2°C. The population of the rhizobacteria and antagonistic fungi in the bio-formulation was 1 cfu g^–1^ × 10^8^cfu g^–1^ and 3 cfu g^–1^ × 10^6^cfu g^–1^.

### Pot Culture Studies on *Fusarium* Wilt Management

The experiment was carried out on tomato cv. Pusa Rohini as described (Shanmugam et al., 2011a, 2015) for a period of 120 days from sowing in a polyhouse using a completely randomized block design with three replicates containing five plants per treatment. For seed treatment, the seeds were surface sterilized with sodium hypochlorite (1%) for 5 min and then soaked in the cell or spore suspensions (3 cfu ml^–1^ × 10^9^ cfu ml^–1^) of the antagonistic bacteria and fungi, and sterile distilled water containing talc-based formulations (20 g^–1^) were applied as seed treatment. After overnight (12 h) incubation at room temperature (28°C ± 2°C) in dark, the suspension was drained off and the seeds were dried under shade for 30 min. Ten seeds were sown in individual 20 cm depth and 20 cm diameter plastic pots containing 6.5 kg steam sterilized soil (121°C, 30 min for 2 consecutive days). Thirty-day-old seedlings were thinned to six per pot and 15 days after thinning, the plants were infected by incorporating 50 ml of conidial suspension (1 × 10^4^ conidia ml^–1^) of a highly pathogenic TOFU-IHBT strain in soil. The cell or spore suspension or talc-based formulation (8 g) containing antagonistic cells was applied two times to the soil at a 45- and 60-day old plant. Seed treatment and soil application with carbendazim (0.1%; 1 g/L) + copper oxychloride (0.25%; 2.5 g/L) were maintained as a fungicide control. Seeds treated with pathogen alone and untreated seeds served as control. The experimental setup was maintained at 20–30°C and 90–95% relative humidity. The severity of wilt incidence and PDI were determined as described earlier. The root and shoot lengths were recorded at the time of harvest. The cultivar developed systemic infection from 4 to 6 weeks of the inoculation.

### Tissue Collection, Enzyme Extraction, and Assays

For the potential biocontrol treatment and the uninoculated control, three plants were uprooted without injury after 6 days of pathogen inoculation and the root samples were processed for gene expression studies. The samples were initially washed under running tap water and then rinsed in distilled water. The samples were homogenized with liquid nitrogen in a prechilled mortar and pestle, and the homogenized root tissues were stored at −80°C. Total RNA was isolated from 100 mg of the frozen samples using Tri-reagent (Invitrogen, Waltham, MA, United States) following the manufacturer’s protocol. cDNA synthesis was carried out with 1 μg of RNA by adding 1 μl of oligo DT, 0.5 μl dNTPs, 2 μl 10X RT buffer, 1 μl Firescript reverse transcriptase, 0.5 μl RNAse inhibitor, and reverse transcription was carried out at 37°C for 30 min and 85°C for 5 min. The amounts of cDNA in the samples were balanced by employing actin (ToActin F 5′-AGGCAGGATTTGCTGGTGATGATGCT-3′ and ToActin R 5′-ATACGCATC CTTCTGTCCCATTCCGA-3′) as a house-keeping gene. Quantitative reverse transcription PCR (RT-PCR) was carried out in a 30 μl reaction volume consisting of cDNA (2 μl), SYBR Green (15 μl), and 100 nM final concentration of defense gene primers (1 μl) (Table 1). The assays were conducted in a (Roche, Basel, Switzerland) Light cycler 480 with an initial denaturation for 180 s at 95°C, 40 cycles of 95°C for 15 s, 60°C for 20 s, and 72°C for 20 s. Each of the samples along with no template control was used in triplicate, and the experiment was done in duplicate.

**TABLE 1:** Primers used in qRT-PCR assay to assess the expressions of defense genes in tomato induced by the biocontrol treatments on challenge inoculation with the *Fusarium oxysporum* f. sp. *lycopersici* strain, TOFU-IHBT.

**Gene/Protein ID**	**Primers**	**Sequences (5′–3′)**
Actin	RTTOACTIN-F	AGGCAGGATTTGCTGGTGATGATGCT
	RTTOACTIN-R	ATACGCATCCTTCTGTCCCATTCCGA
Endochitinase VIR1	RTVIR-F	ACGATCATTCCAGAACACCG
	RTVIR-R	GCCTTGTAATCCCAGATACCG
*G6pd/*XP_001657190	RTG6PD-F	GTATCAACACCTTCCACCCC
	RTG6PD-R	GATCTTGTACACGCCTAGAGG
Araport:AT1G56190	RTPGK-F	AGATGAACTGCGACCTGATC
	RTPGK-R	AACTCCCACACGATGCTG
*NDUS8/*EC:7.1.1.2	RTNDIS-F	CTGAGCAAGGACTGGAACAG
	RTNDIS-R	GTACTTCAGGGTCAGCATCAG

### Biocontrol Under Field Conditions

The carrier-based formulations of the best performing individual strains and strain mixtures identified from polyhouse studies were further assessed for their performances by the two field experiments at the host institute, an endemic location for the vascular wilt disease. Both the field experiments on tomato cv. Pusa Rohini were carried out from mid-October to mid-March during 2018 and 2019. The soil texture in the field was clay loam with a pH of 5.8 and an EC of 0.095 mmhos cm^–1^. The organic matter content was 4.07%, and the amount of N:P:K was 222.66:39.73:562.01 kg ha^–1^). The tomato seeds were soaked overnight in a water solution made with the talc-based formulations (20 g L^–1^) of the biocontrol consortia. The seeds were separated from the biocontrol suspension by draining off the latter, dried overnight under aseptic conditions, and then planted in 20 cm diameter plastic pots after drying overnight. One-month-old seedlings of 16 numbers were transplanted in plots of 6′ (1.8 m) × 4′ (1.2 m) size at 40 cm × 40 cm spacing accommodating four rows of four seedlings each. The plots were replicated three times and arranged in a randomized block design. The formulation was also applied to the soil (200 g bed^–1^) three times at 45, 60, and 75 days of planting by mixing with an equal quantity of farmyard manure. Copper oxychloride-0.25% + carbendazim-0.1% were applied as seedling treatment and soil application, and the combination was used as a fungicide control. Seedlings treated with formulation slurry followed by soil application and untreated seedlings served as control. Agronomic practices, such as irrigation, fertilization, and other cultural practices like weeding and hoeing were followed as a recommendation to the farmers. Ten plants were chosen at random from each plot and assessed for natural incidence (PDI) of yellows 65 days after sowing. Fruits were collected regularly and weighed, and 180-day cumulative yields were calculated as fruit number and weight per treatment. The yield parameters on shoot length and root length were recorded at the time of harvest. The *Fusarium* population in the rhizospheres of inoculated plants was determined at 0- (control), 30-, 60-, and 90-day post-inoculation. Isolation was made by a serial dilution plate technique on Fo-G1 (Nishimura, 2007), and the fungal number was determined by the plate counting method. The isolates were purified and checked for pathogenicity as described earlier.

### Data Analyses

The NTSYS.PC (Numerical Taxonomy System Applied Biostatistics, Setauket, NY, United States) computer program was employed for the analysis of the fingerprints. The data (presence or absence of band) were introduced in the form of a binary matrix, and a pairwise similarity matrix was constructed using the Jaccard coefficient. The dendrogram was generated by the Unweighted Pair-Group Method with Arithmetical Averages (UPGMA) method using the NTSYS program. For data analysis, the presence or absence of a given band was scored as 1 and 0, respectively. The pot culture experiments were repeated two times with similar results. Hence, one representative trial is reported. Statistical analyses for ANOVA were conducted using the IRRISTAT version 92-1 program (IRRI, 1992) developed by the biometrics unit at the International Rice Research Institute, the Philippines. Differences between the mean values of the treatments were determined using an LSD test at a 0.05 probability level.

## Results

### Identification of the *Fusarium* Isolates

**FIGURE 1:**
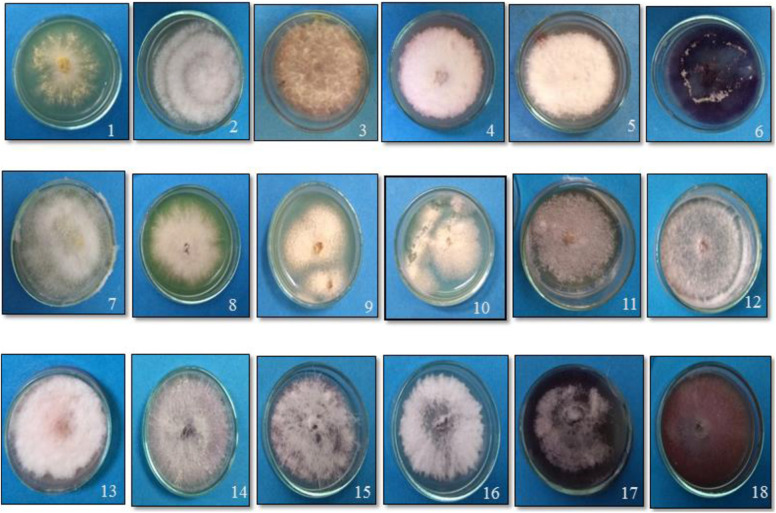
Growth of the putative *Fusarium* isolates of tomato on potato dextrose agar medium. (1) TOFOL-IHBT; (2) TOFU-2-SOGHI; (3) TOFU-3-B.GHAT; (4) TOFU-IHBT; (5) TOXX- POT-1; (6) TOXX- POT- 2; (7) TOFU-KOTBEJA-1; (8) TOFOL-CBE; (9) TOFS-CPCT-2; (10) TOFS-3-CPCT; (11) TOFU-MM; (12) TOFS-MU; (13) TOFU-4-CPCT; (14) TOFU- 6-CPCT; (15)TOFU-TISSA-4; (16) TOFS-IIVR; (17) TOFU-SN; (18) TOFU-5.CPCT.

*Fusarium* wilt of tomato is universally present to the extent of 17–32% in the tomato growing regions surveyed in the states (Uttar Pradesh and Tamil Nadu) of India. Based on the morphological characterization of the fungal isolates on potato carrot agar, only the association of *F. oxysporum*. (Nelson et al., 1983) was observed in the infected stem vascular tissues of Tamil Nadu. On the contrary, one sample of Uttar Pradesh yielded *Fusarium solani* (FS) in addition to Fol. Among the laboratory collections, except for the two samples of Himachal Pradesh and one sample of Delhi, Fol was predominant (Table 2). On PDA, Fol displayed white aerial mycelium tinged with loosely floccose, delicate, purple pigmentation with occasional bluish violet sclerotial bodies (Figure 1). Conidiophores are branched and unbranched monophialides and are short with abundant microconidia. The microconidia are largely single-celled, kidney or oval-shaped, and are produced in false heads, which distinguish the fungus from the closely related *Fusarium* species. Macroconidia are abundant, thin-walled and delicate, slightly curved with a foot-shaped basal cell, and an attenuated apical cell, 4–6 septate. Chlamydospores appear singly or in pairs. The growth of FS on PDA was rapid with white and dense aerial mycelium with abundant macroconidia and microconidia. Microconidia were lemon to pear-shaped with 0–1 septum. Macroconidia were sickle-shaped, and their basal cells were distinctly notched or foot-shaped. Like Fol, the chlamydospores also appear singly or in chains. In the artificial inoculation assays to prove pathogenicity, the plants inoculated with either of the fungal species on incubating at 85% humidity and 28°C developed vascular wilt symptoms in 7–14 days, and the symptoms were similar to those of the diseased tomato plants. The diseased plant tissues on reisolation yielded the fungal species displaying typical conidial and colony morphologies characteristic to the respective inoculated fungus. The asymptomatic and uninoculated control plants did not yield any fungus. Though all the 14 Fol and four Fs isolates were pathogenic, one isolate, TOFU-IHBT, characterized as Fol and could inflict vascular wilt symptoms (100 per cent PDI) within 3 weeks is identified as a highly pathogenic one and used for pot culture studies on biocontrol. In sequencing the ITS region of the 18 isolates to confirm their identity, the Fol and Fs isolates displayed 99–100% sequence identities to those of the strains, WZ-176 of *F. oxysporum* (GenBank Accession No. MN856310.1) and CP1 of FS (GenBank Accession No. MH729019.1) from the databases. The identity of the isolates was further confirmed by sequencing the *tef* gene. Both the isolates of Fol and Fs displayed 99–100% sequence identities to those of the strains, A117-W-YY of *F. oxysporum* (Accession MT313924.1) and NRRL 22639 of FS (Accession No. MK818416) from the databases. Thus, the identity of all the 18 isolates was established based on colony characteristics by proving Koch’s postulates and by the analyses of the ITS and *tef* sequences.

**TABLE 2:** List of *Fusarium* isolates of tomato used to study the genetic diversity.

**Code**	**Place of collection**	**Identity (ITS)**	**GenBank**
TOFOL-IHBT	IHBT, Himachal Pradesh	*Fusarium oxysporum*	HM484352.1
TOFU-2- SOGHI	Shoghi, Himachal Pradesh	*Fusarium oxysporum*	MT994796.1
TOFU-3- B.GHAT	Bararighat, Himachal Pradesh	*Fusarium oxysporum*	MT994797.1
TOFU-IHBT	IHBT, Himachal Pradesh	*Fusarium oxysporum*	MW290472.1
TOXX- POT-1	Pot, Himachal Pradesh	*Fusarium oxysporum*	MW248312.1
TOXX- POT- 2	Pot, Himachal Pradesh	*Fusarium oxysporum*	MW341445.1
TOFU-KOTBEJA-1	Kotbeja, Himachal Pradesh	*Fusarium oxysporum*	MW046058.1
TOFOL-CBE	CBE, Tamil Nadu	*Fusarium oxysporum*	GU048878.1
TOFS-CPCT-2	CPCT, Delhi	*Fusarium oxysporum*	KX230440.1
TOFS-3-CPCT	CPCT, Delhi	*Fusarium oxysporum*	MT994795.1
TOFU-MM	MM, Himachal Pradesh	*Fusarium oxysporum*	MW248313.1
TOFS-MU	Mau, Uttar Pradesh	*Fusarium oxysporum*	MW250868.1
TOFU-4-CPCT	CPCT, Delhi	*Fusarium oxysporum*	MT994794.1
TOFU-6-CPCT	CPCT, Delhi	*Fusarium oxysporum*	MT994798.1
TOFU-TISSA -4	Tissa, Himachal Pradesh	*Fusarium solani*	MW341446.1
TOFS-IIVR	IIVR, Uttar Pradesh	*Fusarium solani*	MW341447.1
TOFU-SN	Solan, Himachal Pradesh	*Fusarium solani*	MT945408.1
TOFU-5-CPCT	CPCT, Delhi	*Fusarium solani*	MW018449.1

### Genetic Diversity of *Fusarium oxysporum* Strains

**FIGURE 2:**
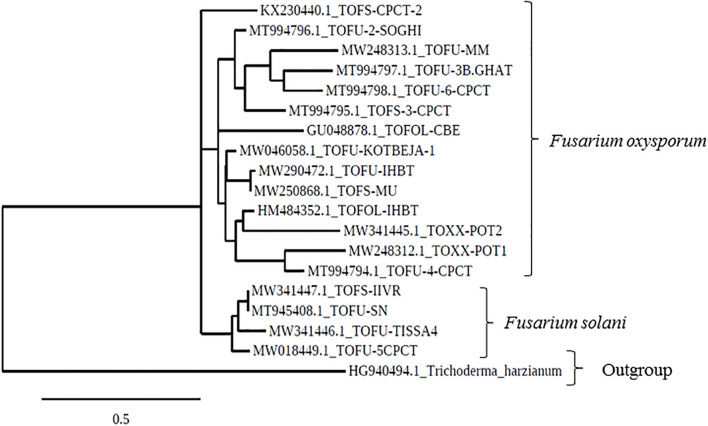
Phylogenetic tree based on ITS gene sequences, drawn using the Maximum likelihood method and showing the relation between *Fusarium* strains.

**FIGURE 3:**
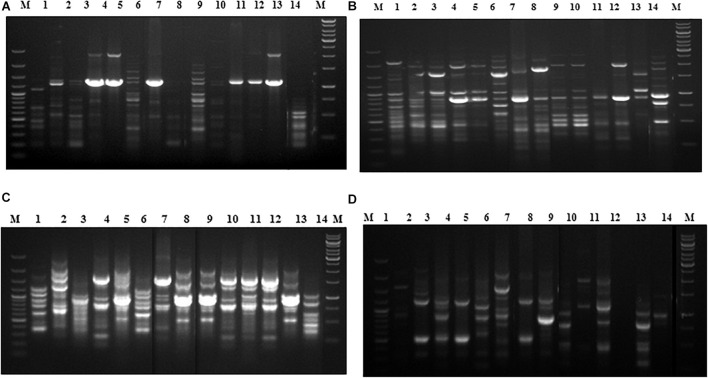
Fingerprinting of *Fusarium oxysporum* strains with ISSR specific primers, **(A)** UBC855, **(B)** UBC 856, **(C)** ISSR 9, **(D)** ISSR 10. Lanes (1) 100 bp plus ladder (Fermentas); (2) TOFOL-IHBT; (3) TOFU-2-SOGHI; (4) TOFU-3 B.GHAT; (5) TOFU-IHBT; (6) TOXX- POTI; (7) TOXX-POT2; (8) TOFU-KOTBEJA; (9) TOFOL-CBE; (10) TOFS-CPCT2; (11) TOFS-3-CPCT; (12) TOFU-XM; (13) TOFS-MU; (14) TOFU-4CPCT; (15) TOFU-6-CPCT; (16) 1 kb ladder (Fennentas).

**FIGURE 4:**
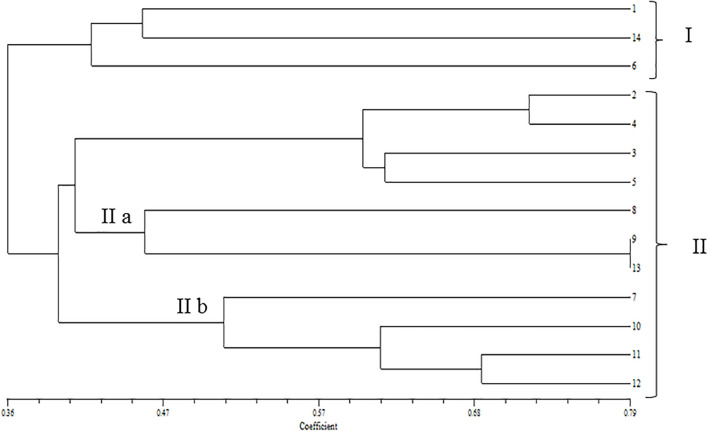
Dendrogram of UPGMA cluster analysis constructed by using DICE similarity coefficients calculated for the *Fusarium oxysporum* strains based on UBC 855; UBC 856; ISSR 9 and ISSR 10. (1) TOFOL-IHBT; (2) TOFU-2- SOGHI; (3) TOFU-3 B.GHAT; (4) TOFU-IHBT; (5) TOXX- POTI; (6) TOXX-POT2; (7) TOFU-KOTBEJA; (8) TOFOL-CBE; (9) TOFS-CPCT2; (10) TOFS-3-CPCT; (11) TOFU-MM; (12) TOFS-MU; (13) TOFU-4CPCT; (14) TOFU-6-CPCT.

In the phylogenetic analyses of the ITS sequences, the strains of Fol and Fs form separate clusters and were grouped along with the reference strains of *F. oxysporum* (Accession No. KT211527.1) and FS (Accession No. KX650831.1), respectively, as a single cluster (Figure 2). The extent of genetic diversity among the strains was further assessed by studying the level of polymorphisms exhibited by the ISSR region. In PCR amplification of genomic DNA of the pathogenic strains, four primers, UBC 855, UBC856, ISSR9, and ISSR10, gave specific, reproducible, and scorable bands with a high percentage of polymorphisms (40–60%). The number of polymorphic bands varied from 15 to 20 with 200–3,500 bp size range. No monomorphic bands were observed in all the primers. Among the primers, only the pattern generated by UBC855 and ISSR10 differentiated the strains of *F. oxysporum* (Figure 3) generating 60% polymorphisms. The Jaccard’s similarity coefficients among the strains varied from 0.36 to 0.79%. The maximum similarity coefficient was observed for the Fol strains and the strains, and TOFS-CPCT-2 and TOFU-4-CPCT showed 100% similarity. In the UPGMA analysis, the genetic relatedness among the Fol strains revealed by the dendrogram (Figure 4) as two major clusters. Cluster I consist of three Fol strains, TOFOL-IHBT, TOXX-POT2, and TOFU-6-CPCT. Cluster II was further divided into two sub-clusters. Sub-cluster IIa consists of seven strains, which are further grouped into two separate clusters comprising four and three strains. The four strains, TOFU-2-SOGHI, TOFU-3B. GHAT, TOFU-IHBT, and TOXX-POT1, exhibited similarity indices of 60–72%. In the latter, though the strains exhibited 45% similarity among themselves, the strains TOFS-CPCT2 and TOFU-4-CPCT exhibited 100% similarity. Four strains consisting of TOFU-KOTBEJA, TOFS-3-CPCT, TOFU-MM, and TOFS-MU formed the second sub-cluster IIb with similarity indices of 50–70%. Interestingly, the clustering did not correlate with the geographic origins of the strains, and the two largest clusters contained strains from all the regions.

### Evaluation of the Microbial Strains for Antagonism Against the Pathogen

**FIGURE 5:**
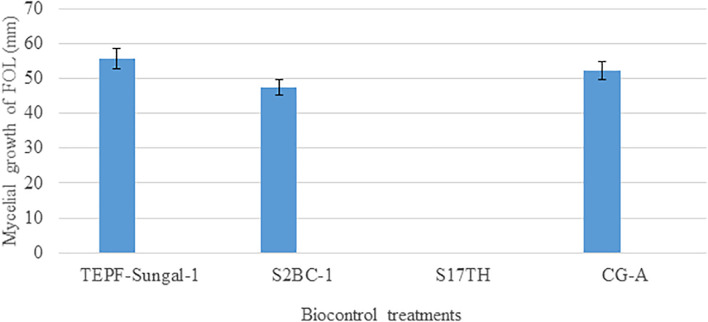
Antagonism of the candidate biocontrol strains against the *Fusarium oxysporum* strain, TOFU- IHBT in dual culture assay. No mycelial growth of the pathogen was observed in the dual culture assay with the *Trichoderma harzianum* strain S17TH. Error bars indicate Standard Deviation (SD).

A dual-culture assay for antifungal activity indicated that the biocontrol strains significantly inhibit the mycelial growth of the pathogen with 37.7–42.6% efficiency over pathogenic control with varying levels of antagonism. Among S2BC-1, TEPF-Sungal-1 and CG-A strains exhibiting the zone of inhibition, S2BC-1 followed by CG-A exhibited a maximum mycelial growth inhibition, displaying 9- and 11.9-mm inhibition zones on co-inoculation. In the TEPF-Sungal-1 treatment, the pathogen recorded a mycelial growth of 55.6 mm with a 34.4% reduction over control. The *Trichoderma* strain S17TH exhibiting mycoparasitic activity was also observed to be highly antagonistic for displaying class I level of antagonism (Figure 5).

### Testing of Compatibility Between the Antagonistic Strains

In the confrontation assays by dual inoculation, none of the antagonistic strains inhibited the growth (data not shown) of the other indicating positive interactions among them. Both the bacterial strains did not show mycelial growth inhibition of S17TH and CG-A in a dual-culture assay. Likewise, S17TH and CG-A did not exhibit mutual antagonism between them. In the co-inoculation assay for the bacterial strains, the strains did not display inhibition zones indicating a lack of antagonism among them. Hence, the strains were selected to develop microbial consortia.

### Polyhouse Evaluation of Selected Antagonists

**FIGURE 6:**
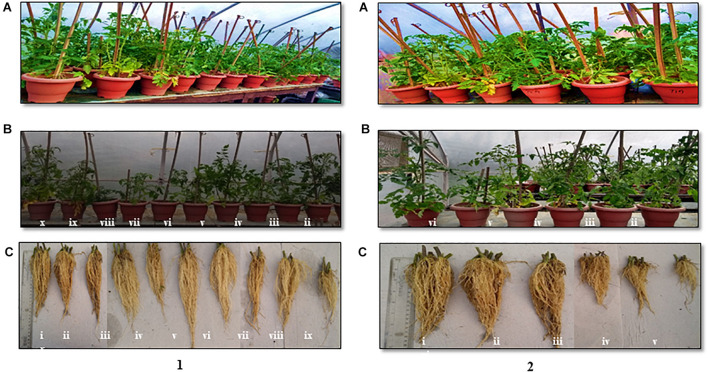
Polyhouse evaluation of the biocontrol agents to manage *Fusarium* wilt of tomato on challenge inoculated with the highly virulent *Fusarium oxysporum* strain, TOFU-IHBT **(A)** overview **(B)** shoot length **(C)** root length. (1) pot experiments with cell suspensions (i) S2BC-1 + TEPF-Sungal-1 + S17TH; (ii) S2BC-1 + S17TH + CG-A; (iii) TEPF-Sungal-1 + CG-A + S17TH; (iv) S2BC-1; (v) TEPF-Sungal-1; (vi) S17TH; (vii) CG-A; (viii) pathogenic (TOFU-IHBT) control; (ix) fungicide (copper oxychloride-0.25%-rcarbendazim-0.1%) control; (x) untreated control. (2) pot experiments with carrier based bioformulations (i) S2BC-1 + TEPF-Sungal-1 + S17TH; (ii) S2BC-1 + S17TH + CG-A; (iii) TEPF-Sungal-l + CG- A + S17TH; (iv) pathogenic (TOFU-IHBT) control; (v) fungicide (copper oxychloride-0.25%-rcarbendazim-0.1%) control; (vi) untreated control.

In the pot experiments, the seed and soil applications of the antagonistic microbes as cell suspensions and carrier-based formulations on the challenge of inoculation with Fol significantly reduced the wilt incidence on the tomato to 41.7–75 and 50–75%, respectively, besides promoting the crop growth to 21.8–55.7 and 49.1–52.3%, respectively, over the pathogenic control treatment. The efficacies of the antagonistic microbes in disease reduction and growth promotion were also significant in recording 13–22.7% efficacies, respectively, in relation to the fungicide treatment (Table 3). Among the antagonistic microbial treatments, in comparison with the individual strains (S2BC-1, TEPF-Sungal-1, S17TH, and CG-A), the application of strain mixtures (S2BC-1 + TEPF-Sungal-1 + S17TH, S2BC-1 + S17TH + CG-A, and TEPF-Sungal-1 + S17TH + CG-A) as cell suspensions and carrier-based formulations significantly reduced the wilt incidence to 25–58.3% and 50–75%, respectively, and increased the plant growth to 39.7–55.7% and 49.1–52.3%, respectively, in comparison to the pathogen control (Table 3). Among the strain mixture treatments, the mixture of TEPF-Sungal-1 + S17TH + CG-A as cell suspensions and carrier-based formulations recorded a minimum wilt incidence (25% and 25%, respectively) with increased plant growth (55.7 and 52.3%, respectively) over the pathogen control (Figure 6 and Table 3). In comparison to the microbial treatments, the disease incidence for the fungicide treatment was comparable (86.1%) to the pathogen control. This treatment recorded a relatively higher plant growth with a 22.7% increase over the pathogen control.

**TABLE 3:** Polyhouse evaluation of biocontrol strains to manage *Fusarium* wilt of tomato.

**Cell suspension treatments**	Parameters*		**Carrier based bioformulation treatments**	Parameters*	
	**RT + ST (cm)**	**Vascular wilt (PDI) of tomato^a^**		**RT + ST (cm)**	**Vascular wilt (PDI) of tomato^a^**
S2BC-1 + TEPF Sungal-1 + S17TH	92.0^abc^	50.0^b^	S2BC-1 + TEPF Sungal-1 + S17TH	86.5^a^	58.3^b^
S2BC-1 + S17TH + CG-A	100.0^ab^	58.3^b^	S2BC-1 + S17TH + CG-A	87.0^a^	58.3^b^
TEPF Sungal-1 + CG-A + S17TH	102.5^a^	25.0^a^	TEPF Sungal-1 + CG-A + S17TH	88.3^a^	25.0^a^
S2BC-1	67.0^de^	58.3^b^	–	–	–
TEPF-Sungal-1	80.2^ed^	50.0^b^	–	–	–
S17TH	89.0^abc^	58.3^b^	–	–	–
CG-A	85.7^bc^	50.0^b^	–	–	–
Pathogenic control	65.8^de^	100.0^c^	Pathogenic control	60.3^c^	100.0^c^
Copper oxychloride-0.25% + Carbendazim-0.1%	58.0^e^	86.1^c^	Copper oxychloride-0.25% + Carbendazim-0.1%	71.2^b^	86.1^c^
Untreated control	58.2^e^	0.0	Untreated control	60.0^c^	0.0
CD (*P* = 0.05)	16.1	23.5	CD (*P* = 0.05)	11.1	21.3

S2BC-1, TEPF-Sungal-1, S17TH, and CG-A refer to the biocontrol strains belonging to Bacillus subtilis (GenBank No. AM268039), *Pseudomonas putida* (GenBank No. MZ363827), *Trichoderma harzianum* (GenBank No. GU048855), and *Chaetomium globosum* (GenBank No. AY429049), respectively; *mean of three replications; the wilt development on each tomato plant was rated as described (Bora et al., 2004): 0 = no symptoms; 1 = <25% of leaves with symptoms; 2 = 26–50% of leaves with symptoms; 3 = 51–75% of leaves with symptoms; and 4 = 76–100% of leaves with symptoms. The percent disease index was calculated as follows: Disease index = (Σ (rating × number of plants rated)/total number of plants × highest rating) × 100; treatment means followed by a common letter(s) are not significantly different from each other by LSD (0.05).

### Role of Host Defense Genes in Biocontrol of *Fusarium* Wilt

**FIGURE 7:**
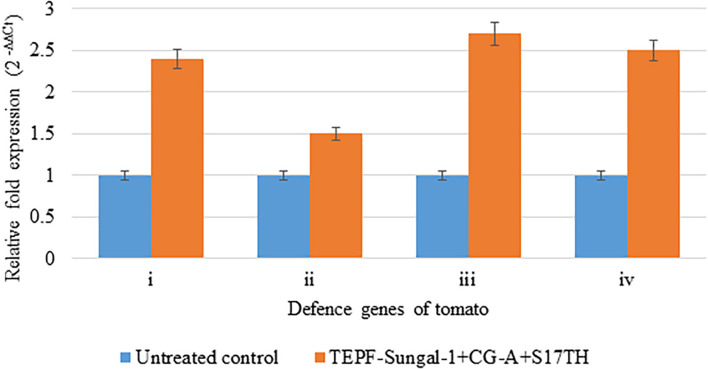
Relative expression of defense genes in the tomato plants cv. Pusa Rohini induced by the potential biocontrol treatment. TEPF-Sungal-1 *(Pseudomonas putida)* + CG-A *(Chaetomium globosum)* + S17TH *(Trichoderma harzianum)* challenge inoculated with the *Fusarium oxysporum* f. sp. *lycopersici* strain, TOFU-IHBT over the untreated control, (i) vzrJ [endochitinase]; (ii) *g6pd* [glucosamine-6-phosphate (Gln-6P) deaminase]; (iii) *PGK* [phosphoglycerate kinase]; (iv) *XDUS8* [NADH dehydrogenase [ubiquinone] iron-sulfur protein 8]. The amount of cDNA in the sample were normalized employing actin as an internal control. Error bars indicate Standard Deviation (SD).

In the quantitative RT-PCR assays to assess the expression of four host genes in response to the potential biocontrol treatment, the seed and soil applications of the strain mixture TEPF-Sungal-1 + CG-A + S17TH during the challenge of inoculation with Fol induced four genes more than the pathogenic control treatment. The biocontrol treatment stimulated endochitinase (*vir1*), glucosamine-6-phosphate (Gln-6P) deaminase (*g6pd*), phosphoglycerate kinase (*PGK*), and NADH dehydrogenase [ubiquinone] iron-sulfur protein 8 (*NDUS8*) genes by 2. 4-, 1. 5-, 2. 7-, and 2.5-fold, respectively, more than those of the pathogen control (Figure 7). Among the genes, the phosphoglycerate kinase followed by the endochitinase showed a maximum upregulation of 2.7–2.4-fold, respectively, in comparison to the control. The induction of the defense genes indicated that the reduction in vascular wilt control by the biocontrol strains can be partly plant-mediated (Figure 7).

### Field Evaluation of Biocontrol Formulations for *Fusarium* Wilt Management

**FIGURE 8:**
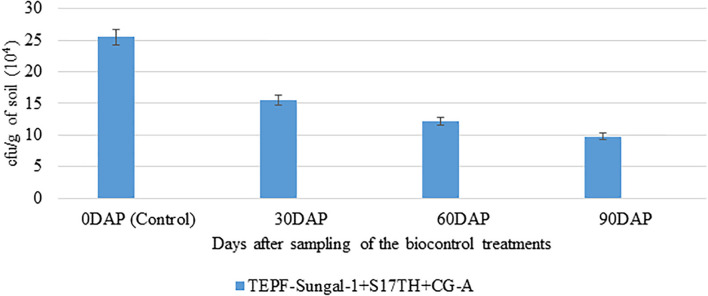
Enumeration of *Fusarium* population in the rhizosphere soils of tomato treated with TEPF-Sungal-1 (*Pseudomonas putida*), S17TH *(Trichoderma harzianum)* and CG-A *(Chaetomium globosum)* under field conditions [VRF, I ARI]; *mean of three replications; the fungal colonies appeared after 3 days of plating on *Fusarium* selective media, Fo-Gl. Error bars indicate Standard Deviation (SD).

**FIGURE 9:**
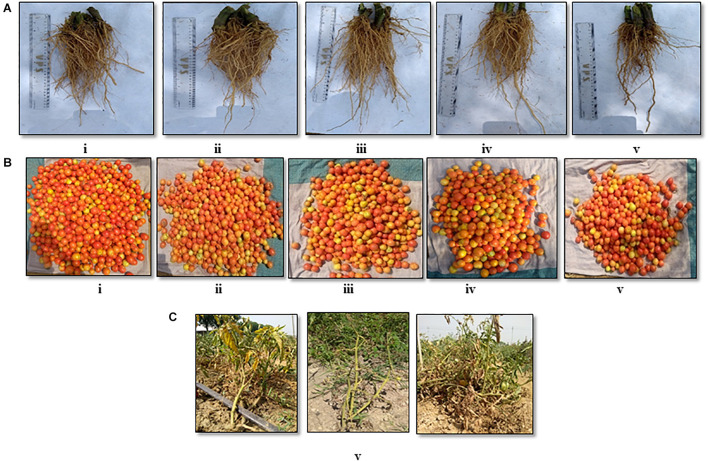
Field evaluation [CPCT, I ARI] of the biocontrol agents to control *Fusarium* wilt of tomato (cv. Pusa Rohini) during harvest for **(A)** root growth, **(B)** fruit yield, **(C)** vascular wilt (i) S2BC-1 + TEPF-Sungal-1 + S17TH; (ii) S2BC-1 + S17TH + CG-A; (iii) TEPF-Sungal-1 + CG-A + S17TH; (iv) fungicide control (copper oxychloride- 0.25% + carbendazim-0.1%); (v) untreated control.

The soil characteristics were analyzed to possess clay loam texture with an organic matter content of 4.07% and N:P:K of 250:50:250 kg/ha. The pH and EC of the soil were analyzed to be 7.5 and 0.3 mmhos/cm^–1^. Both the field trials in 2018–2019 and 2020 indicated that the wilt incidence is low (41.7 and 33.3%) in the microbial treated plots relative to the untreated control. Besides the microbial treated plots of 2018–2019 and 2020 also recorded higher fruit yields of over 32.4 and 105%, respectively, over the untreated control. Among the microbial consortia treatments, the strain mixture, TEPF-Sungal-1 + CG-A + S17TH, performed better than the untreated control. The strain mixture produced lesser wilt incidence with a 54.5 and 60% mean reduction over the untreated control, and the performances were even better than those of the fungicide treatment (18.2%). Besides the treatment also increased the fruit yield by 30.25% over the control. Interestingly, the untreated control recorded a lower fruit yield (34.5 and 15.3 kg) and was followed by the fungicide control (40.4 and 21.9 kg). No pathogenic *Fusarium* population was recorded in the rhizosphere soils treated with the strain mixture, whereas the *Fusarium* population was denser in the rhizospheres of the untreated control plots (Table 4 and Figures 8, 9) and was pathogenic.

**TABLE 4:** Field evaluation of biocontrol agents for management of vascular wilt of tomato.

**Treatment**	Growth and yield parameters*					
	**Trial I-2018-2019 (CPCT)**			**Trial 1I-2019-2020 (VRF)**		
	**Root + shoot length (cm)**	**PDI**	**Fruit weight (kg)**	**Root + shoot length (cm)**	**PDI**	**Fruit weight (kg)**
S2BC-1 + TEPF-Sungal-1 + S17TH	112.1^a^	58.3^c^	44.8^a^	118.1^ab^	50.3^ab^	30.7^a^
S2BC-1 + S17TH + CG-A	112.0^a^	50.0^b^	43.3^a^	126.1^a^	41.7^ab^	29.6^a^
TEPF Sungal-1 + CG-A + S17TH	113.2^a^	41.7^a^	45.6^a^	126.7^a^	33.3^a^	31.3^a^
Copper oxychloride-0.25% + Carbendazim-0.1%	109.2^a^	75.0^d^	40.4^b^	110.8^b^	66.7^bc^	21.9^b^
Untreated control	78.7^b^	91.7^cd^	34.5^c^	75.8^c^	83.3^c^	15.3 ^c^
CD (*P* = 0.05)	9.6	10.8	6.5	12.9	29.0	6.1

S2BC-1, TEPF-Sungal-1, S17TH and CG-A refers to the biocontrol strains belonging to *Bacillus subtilis* (GenBank No. AM268039), *Pseudomonas putida* (GenBank No. MZ363827), *Trichoderma harzianum* (GenBank No. GU048855) and *Chaetomium globosum* (GenBank No. AY429049), respectively; *mean of three replications; the wilt development on each tomato plant was rated as described (Bora et al., 2004); 0 = no symptoms; 1 ≤ 25% of leaves with symptoms; 2-26-50% of leaves with symptoms; 3 = 51–75% of leaves with symptoms; 4 = 76–100% of leaves with symptoms. The per cent disease index was calculated as follows: Disease index = [Σ (rating × number of plants rated)/Total number of plants × highest rating] × 100; treatment means followed by a common letter(s) are not significantly different from each other by LSD (0.05).

## Discussion

Globally, fungal vascular wilt of tomato is known to be caused by the three different species of *Fusarium*, Fol, Fs, and *F. oxysporum* f. spp. *radicis lycopersici* (Kouki et al., 2012). However, in India, *Fusarium* wilt of tomato is caused by either Fol or FS (Chandra et al., 1983; Shanmugam et al., 2011a). We earlier reported the association of Fol with vascular wilt in the selected locations of the states (Himachal Pradesh and Delhi) of India (Sidharthan et al., 2019). In this study, we further studied the etiology of the disease in more tomato growing regions of the country at selected locations of north and south India. The results indicated that the disease is predominated by Fol, and the association of FS has been limited to select locations in the newly surveyed regions. Though mixed infections due to Fol and Fs have been reported globally (Abdesselem et al., 2016) in India, no mixed infections have been reported (Sidharthan et al., 2019). In assessing the genetic diversity of 18 isolates of the two species representing the four Indian states using ITS sequences, the isolates of either of the species did form a distinct group. Recently, based on the molecular phylogenetic analysis of *Fusarium* species associated with tomato, Imazaki and Kadota (2015) revealed Fs as a species complex distinct from that of Fol. In further analyses of the Fol population by the unweighted pair group method with arithmetic averaging using amplicons of ISSR primers, which do not require prior gene sequence information, the strains distinguished themselves into two major clusters and the level of clustering among the isolates of *F. oxysporum* clearly revealed a greater genetic variability within the population, indicating evolutionary adaptation, which may help in the survival of the species with a change in the environment (McDonald and Linde, 2002; Chandra et al., 2008; Sharma et al., 2014). A high genetic diversity of *Fusarium* spp. infected tomato had earlier been reported (Edel-Hermann et al., 2011). Besides genetic polymorphism, no correlation with the geographical delimitation has been observed in the dendrogram developed for the ISSR profiles. No correlation between the genetic diversity and geographic origins of pathogen isolates had been observed for *Fusarium* species infecting several crops (Weller, 1988; Najafiniya and Sharma, 2011). Such a lack of correlation indicates that local ecological niches might be influencing more than the geographical isolation.

Plants living in close association with microbes and rhizosphere soil are identified to be a major microbial reservoir for being influenced by the secretions of roots (Kennedy and de Luna, 2005). Plant health can be improved by engineering the soil microbiota through the host, introducing microbes into the soil or plants and through the manipulation of native microbes by the modification of agricultural practices. The introduction of microbes into the soil for improving crop productivity under field conditions has mostly been accomplished by using single biocontrol agents (Larkin and Fravel, 1998). However, a majority of the strains fail to perform satisfactorily in the field with limited reproducibility upon testing at different locations. A possible reason for the low success and limited reproducibility at different locations could be that the introduced microbial inoculant competes less efficiently with the local microbial community, whose members are extensively diverse and have been well adapted to the environment (Compant et al., 2019). The use of a microbial consortium is suggested as a potential alternative approach for the uncertainty experienced in biocontrol under field conditions. We earlier demonstrated the usefulness of microbial consortia consisting of varying combinations of plant growth-promoting microbes (PGPMs), *Bacillus, Pseudomonas*, and *Trichoderma* in controlling serious soilborne diseases of gladiolus (Shanmugam et al., 2011b) and ginger (Shanmugam et al., 2013) under field conditions. In this study, we tested the microbial consortia consisting of these PGPMs in combination with *C. globosum*, CG-A, a potential fungal biocontrol strain but relatively less explored for disease control under field conditions. The biocontrol potential of CG-A has earlier been demonstrated for the control of diseases in crop plants under polyhouse (Sibounnavong et al., 2011) and field conditions (Cullen et al., 1984). The strains constituting the consortia were carefully selected in such a way that they express different biocontrol traits besides being compatible among themselves. Though the rhizobacterial strains, S2BC-1 and TEPF-Sungal-1, and the *T. harzianum* strain, S17TH were reported to produce lytic enzymes (Shanmugam et al., 2011a,b, 2013; Shanmugam and Kanoujia, 2011), the CG-A strain produced antibiotics, chaetoglobosin, and chaetomin (Aggarwal et al., 2004, 2007). The strains constituting the consortia were also ensured to exhibit growth over a wide range of temperatures ranging from 7°C ± 2°C to 45°C ± 2°C under controlled conditions. In the *in vitro* dual-culture assays, the time of inoculation of the bacterial and fungal biocontrol strains was selected in such a way to favor the pathogen and less conducive to the biocontrol strains. Accordingly, as standardized earlier (Shanmugam and Kanoujia, 2011; Shanmugam et al., 2013) and the bacterial biocontrol strains are fast growing than the fungal biocontrol strains, they were inoculated in the plates after 48 h of pathogen inoculation. On the contrary, the fungal biocontrol strains were co-inoculated with the pathogen since they exhibited a relatively slower growth than the bacterial biocontrol strains but a similar growth to that of the fungal pathogen. The growth rate of each of the strains were standardized earlier in monocultures. To increase the efficiency of the microbial consortia in biocontrol, the microbial strains and their combinations were effectively formulated using a talc-based formulation that could sustain the microbial populations over a period of time during storage (Shanmugam et al., 2013). Accordingly, in the polyhouse studies, the talc-based formulations of the biocontrol treatments, which inhibits Fol, were equally effective like the cell suspensions and were even better than the fungicidal mixture in promoting the crop growth and reducing vascular wilt incidence over the control treatment to significant levels despite the challenge of inoculation with the pathogen. Interestingly, in both the experiments, the application of the mixture of S2BC-1 (*B. subtilis*) + TEPF-Sungal-1 (*P. putida*) as a seed dressing and also a soil application recorded a maximum plant growth promotion and minimum disease incidence in comparison to the uninoculated control. The efficacy of the best performing bioconsortium was further tested in the field at two locations, IARI-CPCT, and IARI-VRF colonized by the two highly pathogenic Fol isolates, TOFU-IHBT and TOFU-CPCT6, for 2 consecutive years. In both the field experiments, the bioconsortium was effective in reducing the vascular wilt incidence to 54.5 and 60% over the untreated control, respectively. Besides the treatment also resulted in improved (32.4 and 45.8%) fruit production higher than the control. Enhanced plant growth and disease suppression by the carrier-based formulations of CG-A have been reported for other diseases (Selvakumar et al., 2001). However, the use of carrier-based formulations of *Chaetomium*-based bioconsortium for disease management in crop plants appears to be the first report. Though many biocontrol agents are potent enough to control plant diseases, only commercial formulations of a few exhibit consistently stable and high efficiency in managing the plant diseases in the field. *Chaetomium*-based bioconsortium demonstrates consistency in its performance across the four experiments in 2 years under the two field conditions.

Understanding the mode of action of biocontrol agents is one of the strategies to improve the success of the biocontrol of plant diseases (Shanmugam et al., 2011b). Multiple antifungal traits known to be produced by the strains constituting the consortium might have accounted for its direct antifungal activity. To understand the role of plant-mediated resistance induced by the biocontrol consortium in vascular wilt control, the induction of candidate defense genes (*vir1, g6pd, PGK*, *and NDUS8*) by the biocontrol treatment was studied. The endochitinase is known to degrade the cell wall components of fungal pathogens (Shanmugam, 2005; Gruber et al., 2011), whereas the *g6pd*, *PGK*, *and NDUS8* genes are involved in various defense reaction pathways of plants (Petriacq et al., 2012, 2013; Rubio et al., 2012). In the polyhouse studies, significant expressions of the four defense genes coupled with the lower vascular wilt incidence and higher plant growth were observed in the bioconsortium treatment over the uninoculated control. Hence, it is speculated that the biocontrol treatment also exerts its antifungal effect through the host plant, and these candidate genes might be involved in the disease suppression. Earlier, the induction of plant defense genes, chitinase, and β-1,3-glucanase leading to decreased disease incidences has been reported (Shanmugam et al., 2011a,b; Shanmugam and Kanoujia, 2011).

It is concluded that the present study has revealed the genetic variability of the *Fusarium* isolates of tomato in the selected tomato growing areas of India, which have been exploited to design and deploy biocontrol agents producing multifunctional traits and occupying different host niches. The potential talc-based formulation of the *Chaetomium-*based bioconsortium can be a component of an integrated disease management system upon commercialization to achieve the goal of practical applications of biocontrol, which are under progress.

## Data Availability Statement

The original contributions presented in the study are included in the article/supplementary material, further inquiries can be directed to the corresponding author/s.

## Author Contributions

GP: experimentation, data analysis, and manuscript drafting. ZH: assistance in *Fusarium* characterization. AKS: assistance in biocontrol experiments. AUS: assistance in qPCR experiments. RA: assistance in biocontrol experiments and manuscript editing. RR: assistance in data analysis. VS: conceptualization, resource, data analysis, and manuscript editing and revision. All authors contributed to the article and approved the submitted version.

## Conflict of Interest

The authors declare that the research was conducted in the absence of any commercial or financial relationships that could be construed as a potential conflict of interest.

## Publisher’s Note

All claims expressed in this article are solely those of the authors and do not necessarily represent those of their affiliated organizations, or those of the publisher, the editors and the reviewers. Any product that may be evaluated in this article, or claim that may be made by its manufacturer, is not guaranteed or endorsed by the publisher.

## References

[B1] Abdel-Azeem A. M. Gherbawy Y. A. Sabry A. M. (2016). Enzyme profiles and genotyping of *Chaetomium globosum* isolates from various substrates. *Plant Biosyst.* 150 420–428. 10.1080/11263504.2014.984791

[B2] Abdesselem S. M. Nisserine H. K. Mebrouk K. Jamal E. H. Jos Eacute S. Eduardo G. (2016). Characterization of *Fusarium oxysporum* isolates from tomato plants in Algeria. *Afr. J. Microbiol. Res.* 10 1156–1163. 10.5897/ajmr2016.8161

[B3] Aggarwal R. (2015). *Chaetomium globosum*: a potential biocontrol agent and its mechanism of action. *Indian Phytopathol.* 68 8–24.

[B4] Aggarwal R. Gupta S. Sharma S. Shukla R. (2014). Development of conventional and real time PCR assay for rapid detection and quantification of a biocontrol agent. *Plant Pathol. J* 46 477–485.

[B5] Aggarwal R. Sharma S. Gupta S. Singh K. B. M. Shanmugam V. (2016). Role of defence enzymes in biocontrol of spot blotch and leaf rust of wheat (*Triticum sp. L*.) by *Chaetomium globosum*. *J. Pure Appl. Microbiol.* 10 2071–2078.

[B6] Aggarwal R. Sharma V. Kharbikan L. L. Renu (2008). Molecular characterization of *Chaetomium spp*. using URP-PCR. *Genet. Mol. Bio.* 31 943–946. 10.1590/s1415-47572008005000014

[B7] Aggarwal R. Tiwari A. K. Dureja P. Srivastava K. D. (2007). Quantitative analysis of secondary metabolites produced by *Chaetomium globosum* Krunze ex Fr. *J. Biol Control* 21 163–168.

[B8] Aggarwal R. Tiwari A. K. Srivastava K. D. Singh D. V. (2004). Role of antibiosis in the biological control of spot blotch (*Cochliobolus sativus*) of wheat by *Chaetomium globosum*. *Mycopathologia* 157 369–377. 10.1023/b:myco.0000030446.86370.14 15281398

[B9] Akbar A. Hussain S. Ullah K. Fahim M. Ali G. S. (2018). Detection, virulence and genetic diversity of *Fusarium* species infecting tomato in Northern Pakistan. *PLoS One* 13:e0203613. 10.1371/journal.pone.0203613 30235252 PMC6147440

[B10] Biswas S. K. Srivastava K. D. Aggarwal R. Prem D. Singh D. V. (2000). Antagonism of *Chaetomium globosum* to *Drechslera sorokiniana* the spot blotch pathogen of wheat. *Indian Phytopathol.* 53 436–440.

[B11] Bora T. Ozaktan H. Gore E. Aslan E. (2004). Biological control of *Fusarium oxysporum* f. sp. *melonis* by wettable powder formulations of the two strains of *Pseudomonas putida*. *J. Phytopathol.* 152, 471–475.

[B12] Chandra S. Raizada M. Gaur A. K. S. (1983). Pathological variability in *Fusarium oxysporum* and *Fusarium solani*. *Indian Phytopathol.* 36 36–40.

[B13] Chandra N. S. Udaya Shankar A. C. Niranjana S. R. Prakash H. S. (2008). Molecular detection and characterisation of *Fusarium verticillioides* in maize (*Zea mays*. L) grown in southern India. *Ann. Microbiol.* 58, 359–367.

[B14] Compant S. Samad A. Faist H. Sessitsch A. (2019). A review on the plant microbiome: ecology, functions, and emerging trends in microbial application. *J. Adv. Res.* 19 29–37. 10.1016/j.jare.2019.03.004 31341667 PMC6630030

[B15] Cullen D. Barbee F. M. Andrews J. H. (1984). *Chaetomium globosum* antagonizes the apple scab pathogen, *Venturia inaequalis*, under field conditions. *Can. J. Bot.* 62 1814–1818. 10.1139/b84-245 33356898

[B16] Divakara S. T. Santosh P. Aiyaz M. Venkata Ramana M. Hariprasad P. Nayaka S. C. (2014). Molecular identification and characterization of *Fusarium spp*. associated with sorghum seeds. *J. Sci. Food Agric.* 94 1132–1139. 10.1002/jsfa.6380 24003016

[B17] Edel-Hermann V. Gautheron N. Steinberg C. (2011). Genetic diversity of *Fusarium oxysporum* and related species pathogenic on tomato in Algeria and other Mediterranean countries. *Plant Pathol.* 61 787–800. 10.1111/j.1365-3059.2011.02551.x

[B18] Gruber S. Kubicek C. P. Seidl-Seiboth V. (2011). Differential regulation of orthologous chitinase genes in mycoparasitic *Trichoderma* species. *Appl. Environ. Microbiol.* 77 7217–7226. 10.1128/AEM.06027-11 21856825 PMC3194852

[B19] Huang C. C. Lindhout P. (1997). Screening for resistance in wild Lycopersicon species to *Fusarium oxysporum* f.sp. lycopersici race 1 and race 2. *Euphytica* 93 145–153. 10.1023/A:1002943805229

[B20] Imazaki I. Kadota I. (2015). Molecular phylogeny and diversity of *Fusarium endophytes* isolated from tomato stems. *FEMS Microbiol. Ecol.* 91:fiv098. 10.1093/femsec/fiv098 26298015

[B21] IRRI (1992). *IRRISTAT User’s Manual. Version 92–1.* Metro Manila: International Rice Research Institute.

[B22] Kennedy A. C. de Luna L. Z. (2005). “Rhizosphere,” in *Encyclopedia of soils in the environment*, ed. Hillel D. (Oxford: Elsevier), 399–406. 10.1016/B0-12-348530-4/00163-6

[B23] Kouki S. Saidi N. Ben Rajeb A. Brahmi M. Bellila A. Fumio M. (2012). Control of *Fusarium* wilt of tomato caused by *Fusarium oxysporum* f.sp. radicis-lycopersici using mixture of vegetable and *Posidonia oceanica* compost. *Appl. Environ. Soil Sci.* 2012:239639. 10.1155/2012/239639

[B24] Larkin R. P. Fravel D. R. (1998). Efficacy of various fungal and bacterial biocontrol organisms for control of *Fusarium wilt* of tomato. *Plant Dis.* 82 1022–1028. 10.1094/PDIS.1998.82.9.1022 30856829

[B25] Lin Y. Fuping L. Shanshan L. Xiao W. Ruixuan Z. Ziqin L. (2012). ISSR analysis of *Fusarium oxysporum* Schl. in Hebei province. *Proc. Environ. Sci.* 12 1237–1242. 10.1016/j.proenv.2012.01.414

[B26] McDonald B. A. Linde C. (2002). Pathogen population genetics, evolutionary potential, and durable resistance. *Annu. Rev. Phytopathol.* 40 349–379. 10.1146/annurev.phyto.40.120501.101443 12147764

[B27] Najafiniya M. Sharma P. (2011). Specific PCR-based marker for detection of pathogenic groups of *Fusarium oxysporum* f.sp. cucumerinum in India. *J. Genet. Eng. Biotechnol.* 9 29–34. 10.1016/j.jgeb.2011.05.009

[B28] Nelson P. E. Toussoun T. A. Marasas W. F. (1983). *Fusarium Species: An Illustrated Manual for Identification.* University Park, TX: Pennsylvania State University Press.

[B29] Nirmaladevi D. Venkataramana M. Srivastava R. K. Uppalapati S. R. Gupta V. K. Yli-Mattila T. (2016). Molecular phylogeny, pathogenicity and toxigenicity of *Fusarium oxysporum* f.sp. lycopersici. *Sci. Rep.* 6:21367. 10.1038/srep21367 26883288 PMC4756691

[B30] Nishimura N. (2007). Selective media for *Fusarium oxysporum*. *J. Gen. Plant Pathol.* 73 342–348. 10.1007/s10327-007-0031-y

[B31] Petriacq P. De Bont L. Hager J. Didierlaurent L. Mauve C. Guerard F. (2012). Inducible NAD overproduction in *Arabidopsis* alters metabolic pools and gene expression correlated with increased salicylate content and resistance to Pst-AvrRpm1. *Plant J.* 70 650–665. 10.1111/j.1365-313X.2012.04920.x 22268572

[B32] Petriacq P. De Bont L. Tcherkez G. Gakiere B. (2013). NAD: not just a pawn on the board of plant-pathogen interactions. *Plant Signal. Behav.* 8:e22477. 10.4161/psb.22477 23104110 PMC3745554

[B33] Rubio M. B. Dominguez S. Monte E. Hermosa R. (2012). Comparative study of *Trichoderma* gene expression in interactions with tomato plants using high-density oligonucleotide microarrays. *Microbiology* 158 119–128. 10.1099/mic.0.052118-0 22075029

[B34] Selvakumar R. Srivastava K. D. Aggarwal R. Singh D. V. (2001). Biocontrol of spot blotch of wheat using *Chaetomium globosum*. *Ann. Plant Prot. Sci.* 9 286–291.

[B35] Shanmugam V. (2005). “Chitinases in defence against phytopathogenic fungi,” in *Crop Protection-Management Strategies*, ed. Prasad D. (New Delhi: Daya Publishing House), 403.

[B36] Shanmugam V. Atri K. Gupta S. Kanoujia N. Naruka D. S. (2011a). Selection and differentiation of Bacillus spp. antagonistic to *Fusarium oxysporum* f.sp. lycopersici and *Alternaria solani* infecting tomato. *Folia Microbiol.* 56 170–177. 10.1007/s12223-011-0031-3 21503737

[B37] Shanmugam V. Chugh P. Sharma P. (2015). Cold-tolerant *Trichoderma* species for the management of Fusarium wilt of tomato plants. *Ann. Microbiol.* 65 543–551. 10.1007/s13213-014-0890-3

[B38] Shanmugam V. Gupta S. Dohroo N. P. (2013). Selection of a compatible biocontrol strain mixture based on co-cultivation to control of rhizome rot of ginger. *Crop Prot.* 43 119–127. 10.1016/j.cropro.2012.08.012

[B39] Shanmugam V. Kanouji N. Singh M. Sing S. Prasad R. (2011b). Biocontrol of vascular wilt and corm rot of gladiolus caused by *Fusarium oxysporum* f.sp. gladioli using plant growth promoting rhizobacterial mixture. *Crop Prot.* 30 807–813. 10.1016/j.cropro.2011.02.033

[B40] Shanmugam V. Kanoujia N. (2011). Biological management of vascular wilt of tomato caused by *Fusarium oxysporum* f.sp. lycopersici by plant growth-promoting mixture. *Biol. Control* 57 85–93. 10.1016/j.biocontrol.2011.02.001

[B41] Sharma M. Nagavardhini A. Thudi M. (2014). Development of DArT markers and assessment of diversity in *Fusarium oxysporum* f.sp. ciceris, wilt pathogen of chickpea (*Cicer arietinum L*.). *BMC Genomics* 15:454. 10.1186/1471-2164-15-454 24912854 PMC4070567

[B42] Sibounnavong P. Charoenporn C. Kanokmedhakul S. Soytong K. (2011). Antifungal metabolites from antagonistic fungi used to control tomato wilt fungus *Fusarium oxysporum* f.sp. lycopersici. *Afr. J. Biotechnol.* 10 19714–19722. 10.5897/AJB11.3343

[B43] Sidharthan K. V. Aggarwal R. Shanmugam V. (2018). Selection and characterization of the virulent *Fusarium oxysporum* f.sp. lycopersici isolate inciting vascular wilt of tomato. *Int. J. Curr. Microbiol. Appl. Sci.* 7 1749–1756. 10.20546/ijcmas.2018.702.212

[B44] Sidharthan V. K. Aggarwal R. Shanmugam V. (2019). “Fusarium wilt of crop plants: disease development and management,” in *Wilt Diseases of Crops and their Management*, eds Bhattacharyya A. Chakraborty B. N. Pandey R. N. Singh D. Dubey S. C. (New Delhi: Today and Tomorrow Printers and Publisher), 519–533.

[B45] Soytong K. Kanokmedhakul S. Kukongviriyapa V. Isobel M. (2001). Application of *Chaetomium* species (Ketomium§) as a new broad spectrum biological fungicide for plant disease control. *Fungal Diver.* 7 1–15.

[B46] Weller D. M. (1988). Biological control of soilborne plant pathogens in the rhizosphere with bacteria. *Annu. Rev. Phytopathol.* 26 379–407. 10.1146/annurev.py.26.090188.002115

[B47] Yang W. Zheng L. Liu H. X. Wang K. B. Wang Y. P. Luo Y. M. (2014). Evaluation of the effectiveness of a consortium of three plant-growth promoting rhizobacteria for biocontrol of cotton *Verticillium wilt*. *Biocontrol. Sci. Technol.* 24 489–502. 10.1080/09583157.2013.873389

[B48] Zhang H. Yang Q. (2007). Expressed sequence tags-based identification of genes in the biocontrol agent *Chaetomium cupreum*. *Appl. Microbiol. Biotechnol.* 74 650–658. 10.1007/s00253-006-0701-2 17221201

