# Corrigendum: Characterization of *Fusarium* Spp. Inciting Vascular Wilt of Tomato and Its Management by a *Chaetomium*-Based Biocontrol Consortium

**DOI:** 10.3389/fpls.2022.861822

**Published:** 2022-02-25

**Authors:** Govindan Pothiraj, Zakir Hussain, Awani Kumar Singh, Amolkumar U. Solanke, Rashmi Aggarwal, Raman Ramesh, Veerubommu Shanmugam

**Affiliations:** ^1^ICAR-Indian Agricultural Research Institute, New Delhi, India; ^2^ICAR-National Institute of Plant Biotechnology, New Delhi, India; ^3^ICAR-Central Coastal Agricultural Research Institute, Goa, India

**Keywords:** *Fusarium* wilt, tomato, *Chaetomium*, *Trichoderma*, PGPR, consortium, biocontrol

In the original article, there was a mistake in Figure 1 as published. The published article contains a duplicated image. The corrected Figure 1 appears below.

**Figure 1:**
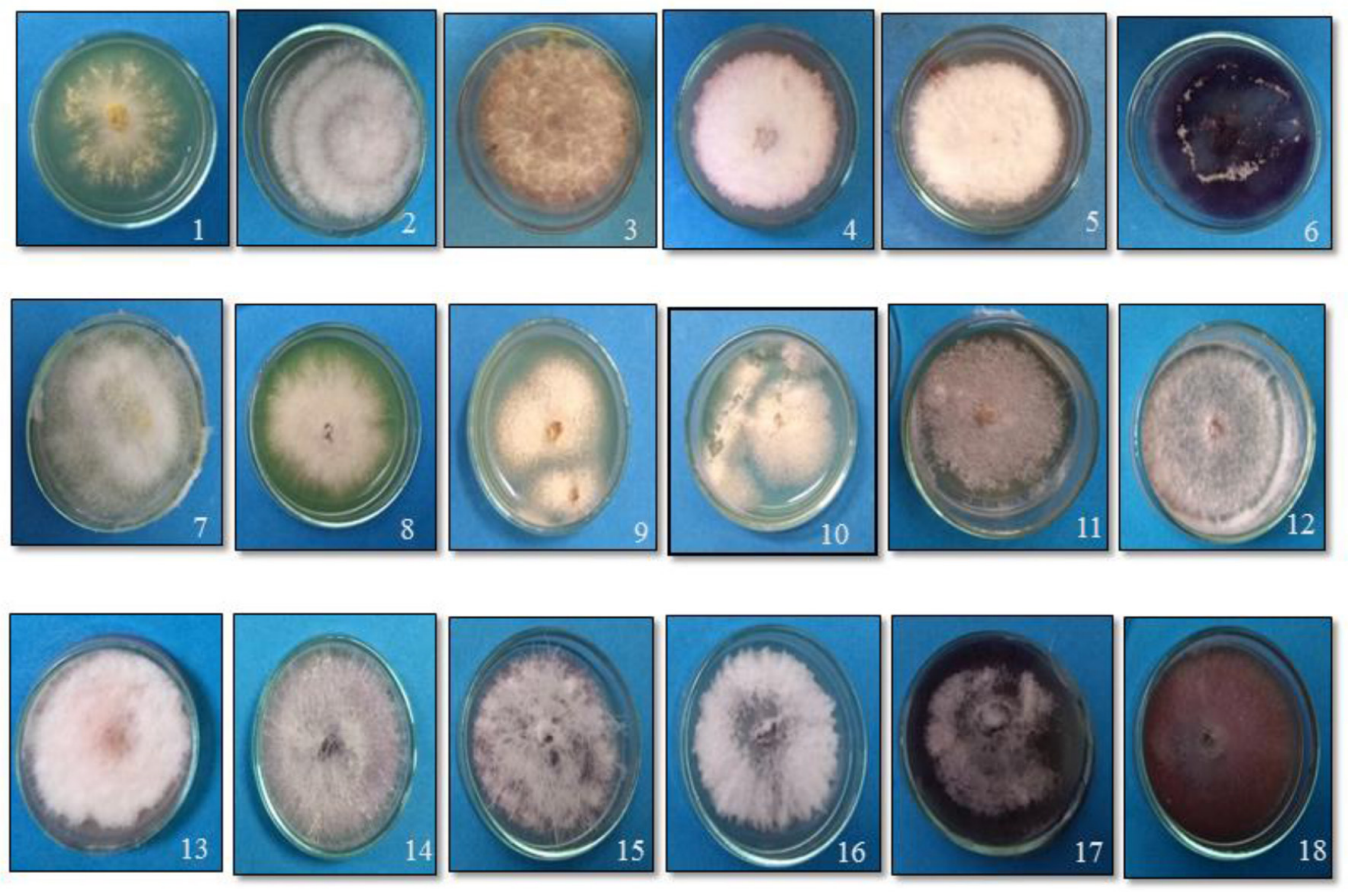
Growth of the putative *Fusarium* isolates of tomato on potato dextrose agar medium. (1) TOFOL-IHBT; (2) TOFU-2-SOGHI; (3) TOFU-3-B.GHAT; (4) TOFU-IHBT; (5) TOXX- POT-1; (6) TOXX- POT- 2; (7) TOFU-KOTBEJA-1; (8) TOFOL-CBE; (9) TOFS-CPCT-2; (10) TOFS-3-CPCT; (11) TOFU-MM; (12) TOFS-MU; (13) TOFU-4-CPCT; (14) TOFU- 6-CPCT; (15)TOFU-TISSA-4; (16) TOFS-IIVR; (17) TOFU-SN; (18) TOFU-5.CPCT.

The authors apologize for this error and state that this does not change the scientific conclusions of the article in any way. The original article has been updated.

## Publisher's Note

All claims expressed in this article are solely those of the authors and do not necessarily represent those of their affiliated organizations, or those of the publisher, the editors and the reviewers. Any product that may be evaluated in this article, or claim that may be made by its manufacturer, is not guaranteed or endorsed by the publisher.

